# Parenteral colistin for the treatment of severe infections by multidrug-resistant Gram-negative bacteria

**DOI:** 10.1186/cc12643

**Published:** 2013-06-19

**Authors:** C Grion, MT Tanita, CM Dantas de Maio Carrilho, JP Garcia, J Festti, LTQ Cardoso, F Chiquetti, MM Kanehissa, CC Branco Lopes, D Blum, V Anami, AR Ruiz, PA Rossatto

**Affiliations:** 1 Hospital Universitário - Universidade Estadual de Londrina, Vila Operária, Londrina, PR, Brazil

## Introduction

This study aimed to describe the use of colistin to treat nosocomial infections caused by multidrug-resistant Gram-negative bacteria and to identify risk factors associated with acute kidney injury and mortality in a single center.

## Methods

This prospective longitudinal study evaluates critically ill patients with nosocomial infections caused by multidrug-resistant Gram-negative bacteria. All adult patients requiring treatment with intravenous colistin (colistimethate sodium) from January to December 2008 were considered eligible for the study. Data include demographics, diagnosis, duration of treatment with colistin and 30-day mortality. Data were analyzed using MedCalc 12 (MedCalc Software, Belgium).

## Results

Colistin was used to treat an infection in 109 (13.81%) of the 789 patients admitted to the ICU. Colistin treatment started an average of 10 (6 to 14.25) days after admission to the ICU. The 30-day mortality observed in these patients was 71.56%. Twenty-four (22.02%) patients developed acute kidney injury, and 11 of these patients required dialysis. The variables independently associated with the presence of acute kidney injury were concomitant use of vancomycin (OR 3.10; 95% CI 1.10 to 8.69; *P *= 0.032) and vasopressors (OR 4.22; 95% CI 1.23 to 14.43; *P *= 0.022). Age (OR 1.03; 95% CI 1.00 to 1.05; *P *= 0.027) and vasopressor use (OR 12.48; 95% 4.49 to 34.70; *P <*0.001) were independent risk factors for death in the logistic-regression model.

## Conclusion

The concomitant use of vancomycin was associated with increased risk of acute kidney injury. Shock and vasopressors were associated with increased risk for acute kidney injury and death.

